# Editorial commentary on the special issue of reproductive medicine

**DOI:** 10.7555/JBR.40.20268003

**Published:** 2026-07-28

**Authors:** Editorial Board

This special issue comprises one review and seven original articles covering diverse topics in reproductive medicine, including the microbiota–metabolite axis in endometriosis, BDE47-induced ferroptosis in spermatocytes, human papillomavirus (HPV) and fertility outcomes in endometriosis, metabolomic biomarkers for azoospermia stratification, gene function validation in male reproduction, endometrial preparation protocols and the vaginal microbiota, bisphenol A (BPA)-induced developmental cardiotoxicity, and diagnostic criteria for gestational diabetes.

Li *et al*^[1]^ reviewed the microbiota of the gut, reproductive tract, and ectopic lesions in endometriosis and proposed a "microbiota–metabolite axis" framework. The authors highlighted how dysbiosis-driven reductions in short-chain fatty acids, imbalances in tryptophan metabolism, elevated β-glucuronidase activity, and increased lipopolysaccharide levels may collectively disrupt immune homeostasis and estrogen metabolism, and highlighted microbiota-targeted interventions as promising non-hormonal therapeutic strategies.

Xu e*t al*^[2]^ investigated BDE47-induced male reproductive toxicity using *in vivo* and *in vitro* models and demonstrated that BDE47 induced testicular CYP3A expression and excessive reactive oxygen species (ROS), which activated the HIF-1α/ALKBH5/ATG12 axis to promote ferritinophagy, resulting in Fe^2^^+^ overload and ferroptotic spermatocyte death. These findings reveal a novel CYP3A-ROS-ferritinophagy-ferroptosis signaling cascade underlying BDE47-induced testicular damage.

Li *et al*^[3]^ conducted a meta-analysis of seven studies and an additional case-control study to evaluate the association between HPV infection and endometriosis risk as well as fertility outcomes. The meta-analysis revealed no significant association between HPV infection and endometriosis risk; however, the case-control study showed that HPV-positive patients had significantly lower postoperative live birth rates, suggesting that HPV infection may impair fertility outcomes in patients with endometriosis.

Zhang *et al*^[4]^ compared letrozole plus human menopausal gonadotropin (HMG) with gonadotropin-releasing hormone agonist (GnRH-a) pretreatment followed by hormone replacement therapy (HRT) for endometrial preparation before frozen embryo transfer among women with endometriosis and characterized vaginal microbiota composition using 16S rRNA sequencing. The live birth rates were comparable between the two protocols. However, the GnRH-a HRT group showed higher relative abundances of several low-abundance taxa, such as *Escherichia-Shigella*, *Limosilactobacillus*, and *Staphylococcus*, indicating that different endometrial preparation protocols may be associated with distinct vaginal microbiota profiles in women with endometriosis.

Fu *et al*^[5]^ generated six CRISPR/Cas9 mutant mouse lines deficient in testis-expressed genes (*Efcab7*, *Tekt3*, *Mlf1*, *Rp1l1*, *Agbl2*, *Tmsb15a*) and evaluated male reproductive phenotypes. None of the mutant lines exhibited overt male infertility, although *Tekt3* deficiency reduced sperm motility without compromising male fertility. These findings suggest that complete or near-complete knockout of the tested murine alleles does not lead to overt male infertility, providing functional evidence for interpreting candidate infertility-associated variants. Nevertheless, these findings do not exclude the possibility of human-specific or allele-specific pathogenic effects.

Wang *et al*^[6]^ performed untargeted metabolomic profiling of seminal plasma from 80 patients with azoospermia and identified candidate biomarker panels that distinguished obstructive azoospermia (OA) from non-obstructive azoospermia (NOA) and further differentiated histological subtypes (Sertoli cell-only syndrome *versus* other subtypes and hypospermatogenesis *versus* maturation arrest), providing a preliminary molecular framework for the preoperative stratification of azoospermia.

Yu *et al*^[7]^ retrospectively analyzed 19152 pregnant women to compare the diagnostic performance of the IADPSG and NICE criteria for gestational diabetes mellitus (GDM). They found that women diagnosed with GDM by the NICE but not by the IADPSG criteria exhibited significantly lower rates of adverse pregnancy outcomes than women diagnosed by the IADPSG criteria. Under current clinical protocols, in which women diagnosed according to the IADPSG criteria receive intervention whereas those meeting only the NICE criteria do not, these findings suggest that the IADPSG criteria more effectively identify women at high risk for multiple adverse pregnancy outcomes, despite classifying fewer women as having GDM in this study.

Jiang *et al*^[8]^ investigated the developmental cardiotoxicity of BPA using human embryonic stem cell-derived cardiac organoids and found that BPA exposure impaired cardiac contractile function and disrupted endothelial network formation, with these functional effects becoming more pronounced at higher concentrations. This study provides *in vitro* evidence that BPA can perturb key processes in early human heart development, underscoring the need for further mechanistic and *in vivo* investigations to clarify its potential contribution to congenital heart defects.

We hope that readers will find this special issue insightful and inspiring.
